# Frizzled2 signaling regulates growth of high-risk neuroblastomas by interfering with β-catenin-dependent and β-catenin-independent signaling pathways

**DOI:** 10.18632/oncotarget.10070

**Published:** 2016-06-15

**Authors:** Karin Zins, Romana Schäfer, Patrick Paulus, Silvia Dobler, Nazak Fakhari, Mouldy Sioud, Seyedhossein Aharinejad, Dietmar Abraham

**Affiliations:** ^1^ Division of Cell and Developmental Biology, Center for Anatomy and Cell Biology, Medical University of Vienna, Vienna, A-1090, Austria; ^2^ Apeiron Biologics AG, Vienna, A-1030, Austria; ^3^ Department of Anesthesiology and Operative Intensive Care Medicine, Kepler University Hospital, Linz, A-4040, Austria; ^4^ Department of Cancer Immunology, Institute for Cancer Research, Oslo University Hospital, Montebello, Oslo, N-0310, Norway; ^5^ Comprehensive Cancer Center (CCC), Medical University of Vienna, Vienna, A-1090, Austria

**Keywords:** Frizzled2, Wnt signaling, neuroblastoma, xenograft model

## Abstract

Frizzled2 (FZD2) is a receptor for Wnts and may activate both canonical and non-canonical Wnt signaling pathways in cancer. However, no studies have reported an association between FZD2 signaling and high-risk NB so far. Here we report that FZD2 signaling pathways are critical to NB growth in *MYCN*-single copy SK-N-AS and *MYCN*-amplified SK-N-DZ high-risk NB cells. We demonstrate that stimulation of FZD2 by Wnt3a and Wnt5a regulates β-catenin-dependent and –independent Wnt signaling factors. FZD2 blockade suppressed β-catenin-dependent signaling activity and increased phosphorylation of PKC, AKT and ERK *in vitro*, consistent with upregulation of β-catenin-independent signaling activity. Finally, FZD2 small interfering RNA knockdown suppressed tumor growth in murine NB xenograft models associated with suppressed β-catenin-dependent signaling and a less vascularized phenotype in both NB xenografts. Together, our study suggests a role for FZD2 in high-risk NB cell growth and provides a potential candidate for therapeutic inhibition in FZD2-expressing NB patients.

## INTRODUCTION

Neuroblastoma (NB) is a childhood embryonal malignancy arising in the peripheral sympathetic nervous system. Half of all children with NB present with features that define their tumors as high-risk with poor overall survival despite intensive therapy [1, 2]. A subset of these tumors are characterized by amplification of the *MYCN* proto-oncogene [2], which occurs in approximately 25% of tumors [3, 4]. However, absence of *MYCN* amplification, stage 4 and an age of 12 or higher at diagnosis are also categorized as high-risk NB suggesting that other factors contribute to high-risk NB [5].

Aberrant regulation of the Wnt signaling pathway is a prevalent theme in cancer biology [6]. Wnt/β-catenin signaling may be of particular relevance to NBs as this program mediates neural crest cell fate and neural stem-cell expansion [7–9]. Activation of the canonical Wnt/β-catenin pathway is triggered by Wnt ligand binding to cell surface receptors of the Frizzled family (FZD) and low-density lipoprotein receptor-related proteins (LRP5 or 6) co-receptors. This leads to the stabilization of cytoplasmic β-catenin and subsequent transcription of Wnt target genes that include *MYC*, *CCND1* (cyclin D1) and others [10, 11]. Importantly, it has been shown that overactivation of the Wnt signaling pathway is due to the overexpression of different FZD receptors in a variety of cancers [12–14]. In high-risk NB without *MYCN* amplification, increased Wnt ligands have been shown together with strongly expressed β-catenin [15].

Besides canonical β-catenin Wnt signaling, β-catenin-independent non-canonical Wnt signaling encompasses those pathways that instead use other modes of downstream signaling [16] and may also affect NB phenotype and growth. In the β-catenin-independent planar cell polarity (PCP) pathway, FZD receptors activate a cascade that involves the small GTPase Rac1 and JUN-N-terminal kinase (JNK), which can lead to changes in cytoskeleton and cell polarity [17]. An important aspect is the crosstalk among canonical and non-canonical signaling pathways. Accordingly, PCP and β-catenin-dependent Wnt signaling are well known to antagonize each other, and inhibiting one will typically upregulate the other. Another β-catenin-independent pathway, the Wnt/Ca^2+^ pathway, can increase the intracellular Ca^2+^ concentration and activate protein kinase C (PKC) [17, 18]. In melanoma cells Wnt5a signaling directs migration and invasion of cells in a PKC-dependent manner [19] and can increase phosphorylated AKT via phosphoinositide 3-kinase (PI3K) [20].

FZD2 is one of the most important receptors in non-canonical Wnt pathways and FZD2 expression is strongly correlated with poor prognosis in several types of cancer [12, 21, 22]. The binding of Wnt5a to FZD2 activates the Wnt/Ca^2+^ pathway in melanoma cell lines [23]. Moreover, Wnt5a/FZD2 signaling has been shown to control cellular migration and invasion in colon cancer [21]. However, in the presence of Wnt3a, FZD2 also activates β-catenin-dependent signaling in pulmonary carcinoma [24]. These reports indicate that FZD2 can activate both β-catenin-dependent and β-catenin-independent signaling. So far no studies have reported the association of FZD2 with NB growth.

In this study, we examine the function of FZD2 and its potential ligands Wnt3a and Wnt5a in *MYCN*-single copy SK-N-AS and *MYCN*-amplified SK-N-DZ NB cell lines. Our data indicate that FZD2 promotes tumor growth in high-risk NBs by regulating β-catenin-dependent and –independent signaling pathways.

## RESULTS

### Expression of FZD receptors and Wnt signaling factors in high-risk *MYCN*-amplified SK-N-DZ and *MYCN*-non-amplified SK-N-AS NB cell lines

We quantified the transcripts of all FZD receptors (FZD1-10) in human *MYCN*-unamplified SK-N-AS and *MYCN*-amplified SK-N-DZ NB cell lines. Quantitative RT-PCR showed that *FZD2* mRNA in both cell lines was the highest, followed by *FZD3*, while expression of other *FZDs* was low (Supplementary information and supplemental Figure 1).

**Figure 1:**
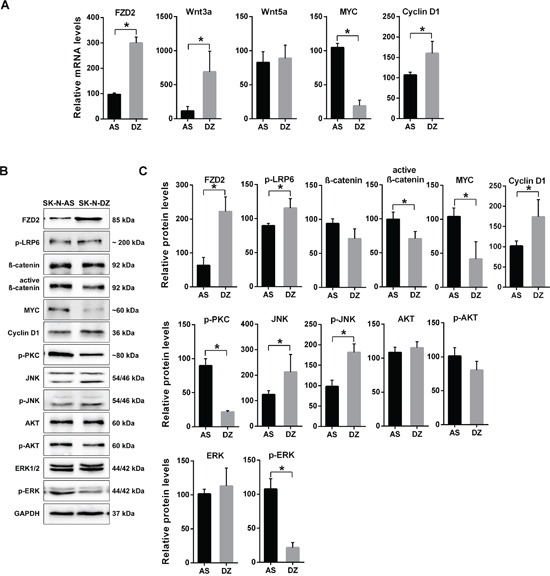
Characterization of gene expression in *MYCN*-unamplified SK-N-AS and *MYCN*-amplified SK-N-DZ NB cells **A.** Graphs show the results of qRT-PCR for FZD2, Wnt3a, Wnt5a, MYC and cyclin D1 performed on RNA from human SK-N-AS (AS) and SK-N-DZ (DZ) NB cells. *FZD2*, *Wnt3a*, *MYC* and *cyclin D1* mRNA expression is different between SK-N-AS and SK-N-DZ cells. **B.** Representative Western blot images and **C.** quantitative determination of protein expression in SK-N-AS and SK-N-DZ cells. Untreated cells were harvested to analyze the levels of FZD2, phospho-LRP6 (LRP6 phosphorylated at Ser1490; p-LRP6), total β-catenin, active β-catenin (non-phospho β-catenin; Ser33/37/Thr41), MYC, cyclin D1, pan-phospho-PKC, (βII Ser660; p-PKC), total JNK, phospho-JNK (JNK phosphorylated at Thr183/Tyr185; p-JNK), total AKT, phospho-AKT (AKT phosphorylated at Ser473; p-AKT), total ERK and phospho-ERK (ERK1/2 phosphorylated at Thr202/Tyr204; p-ERK) by Western blotting. Comparison of protein expression profiles between SK-N-AS and SK-N-DZ cells revealed differences in basal expression and activation levels of investigated signaling proteins. Graphs represent the mean of 3 independent experiments ± SD (* *P* < 0.05).

Next, we investigated the mRNA expression of *FZD2* and its potential activators *Wnt3a* and *Wnt5a* in both NB cell lines. SK-N-DZ cells expressed significantly higher *FZD2* and *Wnt3a* levels, whereas *Wnt5a* was expressed at roughly comparable amounts between SK-N-AS and SK-N-DZ cells (Figure 1A). Notably, expression of the canonical Wnt signaling pathway target *MYC* was significantly higher in *MYCN*-unamplified SK-N-AS, while mRNA of *cyclin D1* was more abundantly expressed in SK-N-DZ cells (Figure 1A).

Basal Wnt pathway activity was examined in the cells using several markers. Low-density lipoprotein receptor-related protein 6 (LRP6) is a key signaling co-receptor for the β-catenin pathway, which is phosphorylated following Wnt binding to FZD2 [17]. Thus, LRP6 phosphorylation, total β-catenin, stabilized active β-catenin, MYC and cyclin D1 were examined by Western blot analysis in both NB cell lines to investigate canonical β-catenin Wnt signaling components. In SK-N-AS cells, FZD2 and phosphorylated LRP6 protein levels were lower compared with SK-N-DZ cells. In contrast, levels of total β-catenin, active β-catenin and MYC were both more abundantly expressed in SK-N-AS cells. SK-N-DZ cells in turn, expressed higher levels of cyclin D1, confirming mRNA findings (Figure 1B and 1C).

Wnt5a signaling activates the PI3K-AKT pathway in melanoma cells [25] and Wnt3a-induced proliferation involves activation of ERK beside Wnt/β-catenin pathway activation in fibroblasts [26]. Therefore, we examined the basal activity of β-catenin-independent non-canonical Wnt signaling components by examining phosphorylation of PKC, JNK, AKT and ERK. SK-N-AS NB cells expressed phosphorylated PKC at higher levels than SK-N-DZ cells. The levels of total and phosphorylated AKT were comparable in both cell lines. In contrast, SK-N-DZ cells expressed higher levels of JNK and phosphorylated JNK (Figure 1B und 1C). The ERK protein level of SK-N-AS cells was comparable to that of SK-N-DZ cells, while phosphorylated ERK was significantly higher in SK-N-AS cells. These data indicate that basal Wnt signaling activity differs in the NB cell lines examined.

### FZD2 regulates β-catenin-dependent and -independent pathways in high-risk NB cell lines

**Figure 2:**
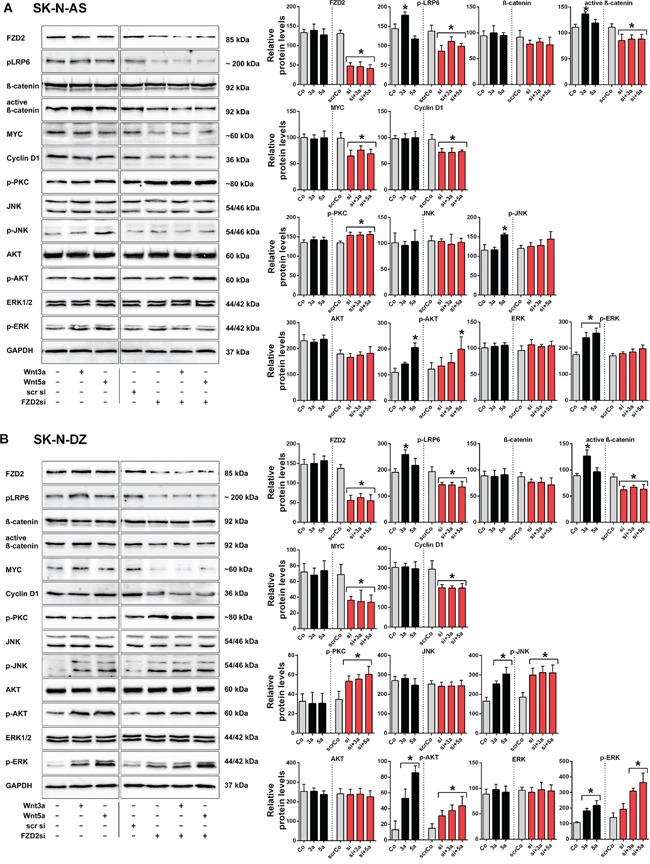
Wnt3a and Wnt5a signal via FZD2 in NB cell lines Representative Western blot images and quantification of immunoblots. SK-N-AS **A.** and SK-N-DZ **B.** NB cells were left untreated (Control; Co) or treated with Wnt3a (3a) and Wnt5a (5a) for 15 min, with or without FZD2 siRNA (si) pretreatment. Nonspecific scrambled siRNA (scr si, scrCo) served as an additional control. Then cells were harvested to analyze the levels of FZD2, phospho-LRP6 (LRP6 phosphorylated at Ser1490; p-LRP6), total β-catenin, active β-catenin (non-phospho β-catenin; Ser33/37/Thr41), MYC, cyclin D1, pan-phospho-PKC, (βII Ser660); p-PKC), total JNK, phospho-JNK (JNK phosphorylated at Thr183/Tyr185; p-JNK), total AKT, phospho-AKT (AKT phosphorylated at Ser473serine-473; p-AKT), total ERK and phospho-ERK (ERK1/2 phosphorylated at Thr202/Tyr204; p-ERK) by Western blotting. Activation levels of signaling factors associated with β-catenin-dependent signaling pathway (p-LRP6, active β-catenin) and β-catenin target genes (MYC, cyclin D1) were reduced below baseline levels in Wnt stimulated and unstimulated NB cells pretreated with FZD2 siRNA in contrast to activation levels of signaling factors associated with β-catenin-independent signaling (p-PKC, p-AKT, p-JNK, p-ERK). Graphs represent the mean of 3 independent replicates ± SD. Asterisks (*) indicate P < 0.05 vs. untreated controls; GAPDH, loading control.

We analyzed whether the FZD2 ligands Wnt3a and Wnt5a may increase or decrease baseline Wnt-signaling activity in NB cells by using recombinant Wnt proteins. In addition, small interfering RNA (siRNA) specific to FZD2 was used to study the effect of FZD2 blockade. For RNA interference experiments, nonspecific scrambled siRNA was used as control. FZD2 siRNA reduced the levels of FZD2 protein by 64% and 59% in SK-N-AS and SK-N-DZ cells, respectively, as compared to cells treated with scrambled siRNA (Figure 2). Determination of the activity of β-catenin-dependent pathways components revealed that Wnt3a but not Wnt5a significantly increased phosphorylation of LRP6 and the level of active β-catenin in SK-N-AS and SK-N-DZ cells. Knockdown of FZD2 decreased phospho-LRP6 and active β-catenin levels in Wnt3a- and Wnt5a-stimulated SK-N-AS and SK-N-DZ cells or unstimulated controls below baseline levels (Figure 2). Total β–catenin levels were not significantly changed compared to baseline levels. Consistent with the results for LRP6 and active β–catenin, MYC and cyclin D1 significantly decreased following knockdown of FZD2. However, no changes were observed by Wnt3a and Wnt5a treatment (Figure 2).

Then, we analyzed activation of factors involved in signaling of β-catenin-independent Wnt pathways. Baseline activity of PKC remained unchanged by Wnt5a and Wnt3a stimulation in both cell types. Of interest, knockout of FZD2 significantly increased phosphorylation of PKC in SK-N-AS and SK-N-DZ cells (Figure 2). Total protein levels of AKT, JNK and ERK remained constant in both cell lines under the observed conditions (Figure 2). Wnt5a increased phosphorylation of JNK in both cell lines, whereas Wnt3a increased phosphorylation of JNK only in SK-N-DZ cells (Figure 2). FZD2 knockdown had no suppressing effect on JNK activity in both cell types (Figure 2). Notably, the basal level of phospho-PKC was 4-fold higher in SK-N-AS cells, whereas the basal phospho-JNK level was 1.9-fold higher in SK-N-DZ cells when comparing protein levels in SK-N-AS and SK-N-DZ cells (Figure 1B and 1C). Stimulation with recombinant Wnt5a increased activation of AKT 1.9-fold in SK-N-AS and 6.6-fold in SK-N-DZ cells, whereas Wnt3a significantly increased AKT activation in SK-N-DZ cells only (4-fold) (Figure 2). No effects on activated AKT levels were observed in FZD2 siRNA treated SK-N-AS cells. Although Wnt3a and Wnt5a increased active AKT levels in FZD2 knockdown SK-N-DZ cells only 2.5-fold and 2.9-fold, respectively, basal active AKT levels increased twofold in these cells (Figure 2B).

To address whether ERK signaling is involved in Wnt/FZD2 signaling, we analyzed phospho-ERK levels. While total ERK-levels remained unchanged, significantly increased activation of ERK was observed in SK-N-AS and SK-N-DZ cells when they were stimulated with Wnt3a (1.4-fold and 1.7-fold, respectively) and Wnt5a (1.5-fold and 2-fold, respectively). Knockdown of FZD2 suppressed Wnt-induced ERK activation in SK-N-AS but not SK-N-DZ cells. Moreover, it increased basal ERK activity in SK-N-DZ cells by 38% (Figure 2B).

These data demonstrated that FZD2 activates the canonical Wnt signaling pathway in NB cells. Further, FZD2 can stimulate and inhibit noncanonical Wnt signaling factors, depending on the NB cell type.

### FZD2 stimulates cell proliferation of human NB cells

**Figure 3:**
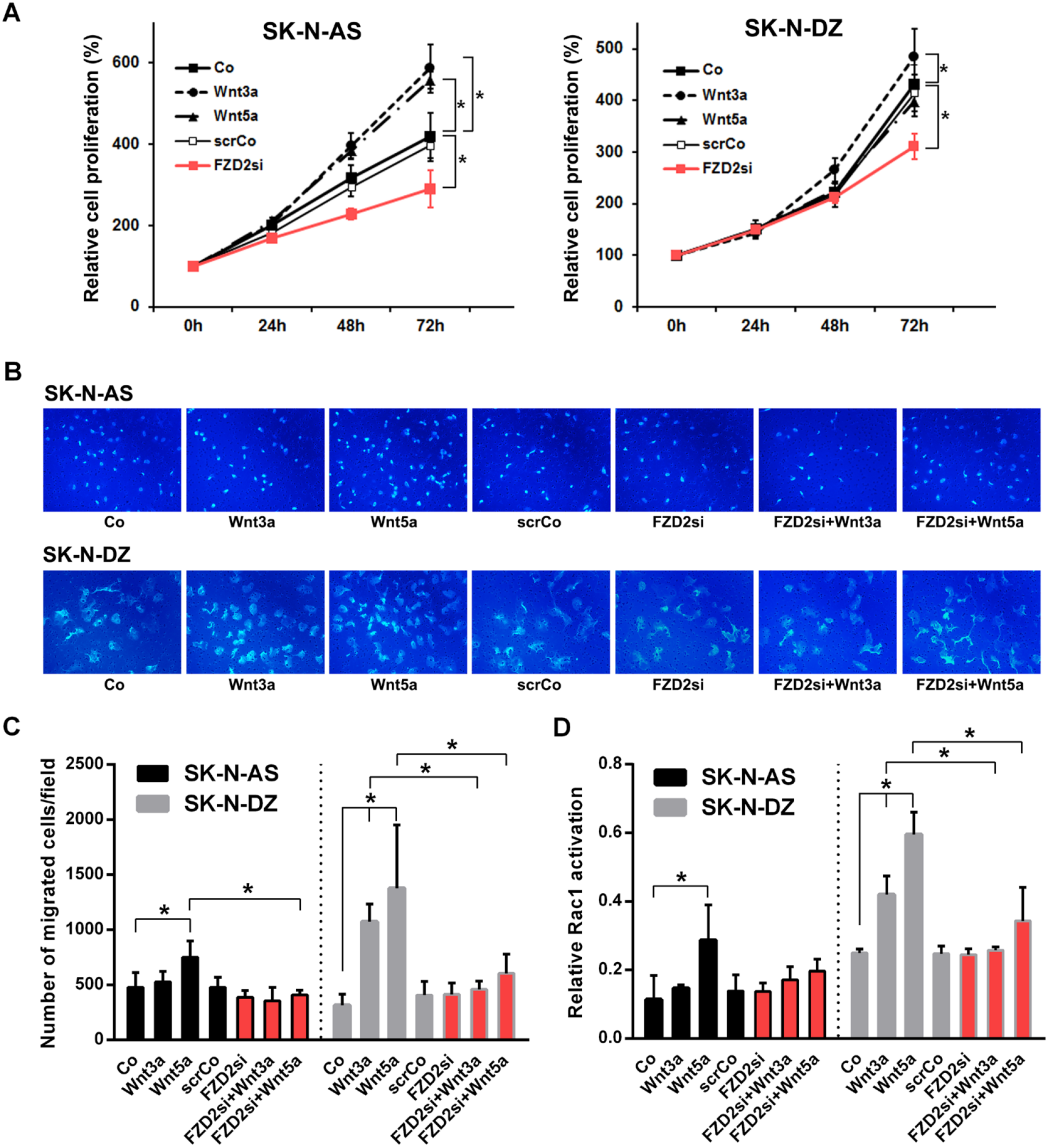
Effects of Wnt3a and Wnt5a stimulation and FZD2 blockade on cell proliferation, migration and Rac1 activity in NB cells **A.** Relative density of cancer cells up to 72 h following stimulation with 100 ng/ml recombinant Wnt3a, Wnt5a or pretreatment with FZD2 siRNA (FZD2si) or nonspecific scrambled siRNA (scrCo) was measured using the WST-1 cell proliferation assay. FZD2 siRNA suppressed NB cell proliferation. Graphs represent the mean of 3 independent experiments ± SD. Asterisks (*) indicate P < 0.04 vs. untreated controls (Co, scrCo). **B.** Representative images of migrated SK-N-AS and SK-N-DZ NB cells from an *in vitro* migration assay are shown (200x magnification). **C.** Quantification of cell migration. NB cells were stimulated with 100 ng/ml recombinant Wnt3a and Wnt5a with or without pretreatment with FZD2 siRNA or scrambled siRNA (scrCo) for 24 h and migrated cancer cells quantified subsequently by *in vitro* migration assays. FZD2 blockade suppressed Wnt-induced migration of NB cells. Graphs represent the mean of 3 independent experiments ± SD. Asterisks (*) indicate P < 0.05 vs. untreated controls (Co, scrCo) and vs. Wnt3a and Wnt5a stimulated cells, respectively. **D.** Rac1 activation in SK-N-AS and SK-N-DZ NB cell lines after stimulation with 100 ng/ml Wnt3a or Wnt5a protein for 30 min with or without FZD2 siRNA or scrambled siRNA (scrCo) pretreatment. FZD2 blockade suppressed Wnt-induced Rac1-activation of NB cells. Graphs represent the mean of 3 independent experiments ± SD. Asterisks (*) indicate P < 0.05 vs. untreated controls (Co, scrCo) and vs. Wnt3a and Wnt5a stimulated cells, respectively.

We then analyzed the effect of FZD2 inhibition on NB cell proliferation. Treatment of unstimulated NB cells with FZD2 siRNA significantly decreased proliferation of SK-N-AS and SK-N-DZ cells compared to untransfected and scrambled siRNA transfected control cells as we assessed by WST-1 assays (*P* < 0.01; Figure 3A). On the other hand, treatment of NB cells with recombinant Wnt3a increased SK-N-AS and SK-N-DZ cell proliferation (*P* < 0.001 and *P* < 0.04, respectively), whereas Wnt5a stimulation increased cellular proliferation of SK-N-AS (*P* < 0.001) but not SK-N-DZ cells as compared with unstimulated cells (Figure 3A). From these experiments, we conclude that FZD2 stimulates the proliferation of both *MYCN*-unamplified and *MYCN*-amplified NB cell lines.

### FZD2 promotes cell migration in NB cells

Wnt5a is also implicated in cancer cell migration [27]. Thus, we set out to evaluate the effects of FZD2 on Wnt3a- and Wnt5a-stimulated NB cell migration in transwell assays. Migration of Wnt3a-stimulated SK-N-AS cells remained unchanged during 48 hours, whereas Wnt5a stimulation significantly enhanced the ability of SK-N-AS NB cells to cross the transwell membrane by 57% relative to control cells (*P* = 0.033). In SK-N-DZ cells, Wnt3a stimulation promoted transwell migration 3.4-fold relative to control cells (*P* = 0.01). Similarly, Wnt5a stimulation promoted migration of SK-N-DZ cells 4.4 fold relative to control cells (*P* < 0.001) (Figure 3B and 3C). In reciprocal experiments, FZD2 knockdown completely suppressed Wnt5a induced migration of SK-N-AS cells (*P* = 0.004 vs. Wnt5a-stimulated cells). FZD2 knockdown also completely suppressed Wnt3a and Wnt5a-induced migration of SK-N-DZ cells (*P* < 0.04 vs. Wnt3a and Wnt5a-stimulated cells, respectively) (Figure 3B and 3C). Overall, we conclude that FZD2 promotes NB cell migration through a process that involves stimulation byWnt5a in SK-N-AS cells and by Wnt3a and/or Wnt5a in SK-N-DZ cells.

### Knockdown of FZD2 suppresses Wnt5a-dependent Rac activation in NB cells

PCP signaling triggers activation of the small GTPase Rac1, which in turn activates JNK, an important factor in cell migration. Moreover, knockdown of FZD2 suppressed the Wnt5a-dependent Rac activation in HeLaS3 cells [28]. To assess the effect of FZD2 on Rac1, we measured Rac1 GTPase activity by G-LISA assay. We first examined Rac1 activation by stimulation with recombinant Wnt3a and Wnt5a in NB cells. Rac1 activity was not affected by Wnt3a treatment in SK-N-AS cells, while Rac1 activity was significantly increased by Wnt3a stimulation in SK-N-DZ cells (*P* = 0.04; Figure 3D). In contrast, Wnt5a significantly upregulated Rac1 activity 2.5-fold in SK-N-AS (P = 0.006) and 2.4-fold in SK-N-DZ cells (P < 0.001) as shown in Figure 3D. FZD2 siRNA treatment caused no decrease of basal Rac1 activity in both NB cell lines. However, it inhibited Wnt5a-dependent Rac1 activation in SK-N-AS cells by 31% and Wnt3a- and Wnt5a-dependent Rac activation in SK-N-DZ cells by 39% and 42%, respectively (P<0.01 vs. corresponding Wnt-stimulated cells) (Figure 3D). These results demonstrate that FZD2 stimulates activation of Rac1 in NB cells.

### FZD2 blockade reduces growth of NB xenografts

**Figure 4:**
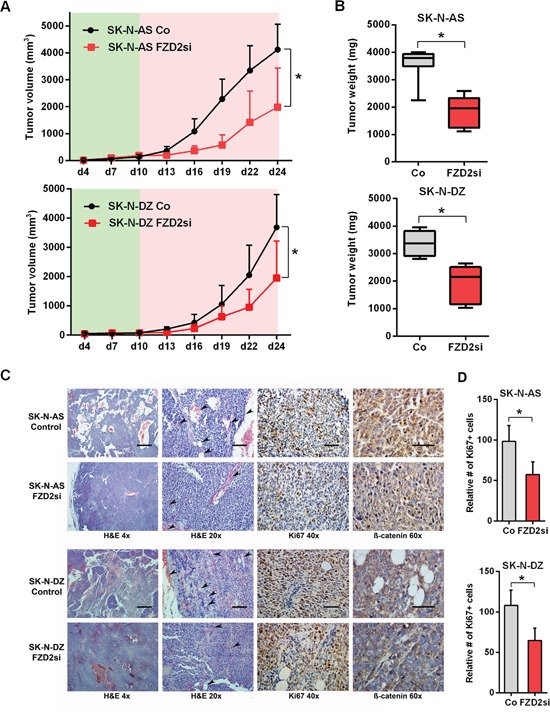
FZD-2 knockdown reduces growth of SK-N-AS and SK-N-DZ xenografts **A.** Tumor volume curves in SK-N-AS and SK-N-DZ tumor xenograft bearing mice treated with FZD2 siRNA (FZD2si) or scrambled siRNA as control (Co). NB cells were injected s.c. into athymic nude mice and tumor growth was monitored in the presence (red shade) or absence (green shade) of siRNA against FZD2 or scrambled siRNA (n = 8 per group). Data are mean ± SD. *, significantly different from control tumors (*P* < 0.05). **B.** Quantification of tumor weight of SK-N-AS and SK-N-DZ NB xenografts on day 24 from mice treated with scrambled siRNA or FZD2 siRNA (n = 8 per group). Box and whisker plots show the mean, quartiles and tenth and ninetieth percentiles of the data. FZD2 blockade significantly suppressed tumor weight of NB xenografts in mice. Data are mean ± SD. *, significantly different from control tumors (*P* < 0.05). **C.** Histology and immunohistochemical analysis of NB xenografts. Representative images of tumor tissue sections from mice treated with FZD2 siRNA or scrambled siRNA on day 24. Left two columns: H&E staining of NBs. SK-N-AS and SK-N-DZ NB xenografts exposed to FZD2 siRNA indicated morphological changes. The scale bar in the left column (4x objective) represents 500 μm and the scale bar in the middle column (20x objective) represents 100 μm. Arrowheads indicate vessels. Third column: NB xenografts stained with the proliferation marker Ki67. The scale bar in the third column (40x objective) represents 50 μm. Right column: NB tumors stained with total β-catenin. β-catenin expression was higher in SK-N-AS tumors. In both, SK-N-AS and SK-N-DZ xenografts, β-catenin staining intensity was reduced in the FZD2 siRNA group. The scale bar in the right column (60x objective) represents 50 μm. **D.** Quantitative histomorphometric analysis of Ki67-positive, proliferating tumor cells. Cellular proliferation was significantly reduced following FZD2 blockade. Data are mean ± SD (n = 8 per group). Asterisks (*) indicate P < 0.05 vs. controls.

To assess the role played by FZD2 in tumorigenesis, we used xenograft models in which SK-N-AS or SK-N-DZ cells were injected subcutaneously into mice. Mice bearing human NB xenografts received five intratumoral injections of FZD2 siRNA or scrambled siRNA as controls. We cycled treatment every three days for two weeks to guarantee continuous reduction of FZD2. At the beginning of treatment on day 10 after tumor cell inoculation, mice developed human tumors of comparable size as measured using a caliper. We observed that tumor growth was significantly slower in the treatment groups compared to the control groups (*P* < 0.01 for both groups on day 24), with a noticeable lag in the exponential growth phase after initiation of therapy on day 10 (Figure 4A). On day 24, at which time the animals were sacrificed, the mean tumor weight was reduced by 49% in SK-N-AS bearing mice treated with FZD2 siRNA (1845 mg ± 546 mg) compared to control mice (3591 mg ± 572 mg; *P* < 0.001) (Figure 4B). Similarly, tumor masses in SK-N-DZ bearing mice (1928 mg ± 667 mg) were reduced by 43% in the FZD2 siRNA group compared with the control group (3366 mg ± 454 mg; *P* < 0.001) (Figure 4B). There were no significant differences in mean body weights between groups (data not shown).

Histological analyses of tumors demonstrated that FZD2 siRNA treated tumors showed morphological changes as readily detected by H&E staining. Tumor cells of SK-N-AS and SK-N-DZ control groups were disorganized and arranged as nests and cords. In contrast, the tumor tissue of FZD2 siRNA treated groups was more dense and homogeneous associated with reduced vessel density (Figure 4C, left two panels). Accompanying the reduction in tumor growth, Ki67^+^ proliferating cells were significantly reduced in both FZD2 siRNA groups by 42% in SK-N-AS, and 40% in SK-N-DZ xenografts (*P* < 0.001; Figure 4C, third panel and 4D). Immunohistochemistry for β-catenin revealed a general higher expression in SK-N-AS neuroblastoma tumors without amplification of *MYCN* as compared to *MYCN*-amplified SK-N-DZ tumors. Blocking FZD2 by siRNA decreased β-catenin staining intensity in SK-N-AS and SK-N-DZ tumors, which then was predominantly found at the periphery of the cells (Figure 4C, right panel).

Taken together, these data suggest that FZD2 blockade reduces tumor growth and tumor cell proliferation associated with decreased β-catenin levels and reduced angiogenesis in both, *MYCN*-unamplified SK-N-AS and *MYCN*-amplified SK-N-DZ NBs.

### FZD2 gene silencing suppresses activation of the β-catenin signaling pathway in NB xenografts

**Figure 5:**
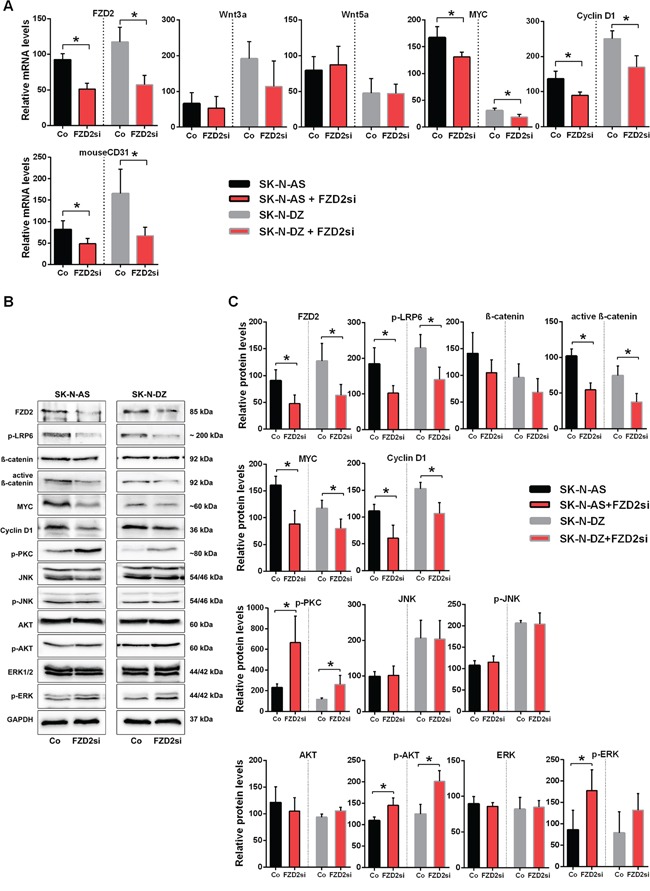
FZD2 blockade interferes with canonical and non-canonical signaling in NB tissue **A.** mRNA expression of SK-N-AS and SK-N-DZ human NB tumor tissue treated with FZD2 siRNA (FZD2si) or scrambled siRNA (Co) on day 24. Graphs show the results of qRT-PCR for human FZD2, Wnt3a, Wnt5a, MYC and cyclin D1 as well as for murine CD31. Data are mean ± SD (n = 8 per group). **B.** Representative Western blot images and **C.** quantification of immunoblots stained with FZD2, phospho-LRP6 (p-LRP6), total β-catenin, active β-catenin, MYC, cyclin D1, pan-phospho-PKC, (p-PKC), total JNK, phospho-JNK (p-JNK), total AKT, phospho-AKT (p-AKT), total ERK, phospho-ERK (p-ERK) antibodies. FZD2 blockade resulted in decreased levels of FZD2, p-LRP6, active β-catenin, MYC and cyclin D1 associated with increased levels of p-PKC, p-AKT and p-ERK in NB tissue. Data are mean ± SD (n = 8 per group). Asterisks (*) indicate P < 0.05 vs. controls.

Along with the tumor suppression following FZD2 siRNA treatment, tissue *FZD2* mRNA levels decreased in both NB xenografts (*P* < 0.02) (Figure 5A). No significant differences were observed for human *Wnt3a* and *Wnt5a* levels in SK-N-AS and SK-N-DZ xenografts following FZD2 siRNA treatment. In contrast, mRNA levels of *MYC*, *cyclin D1*, and the endothelial cell marker *CD31* (murine) were significantly downregulated following FZD2 blockade in both NB xenografts (*P* < 0.05; Figure 5A). Consistently, protein levels of FZD2 and of activated phospho-LRP6 were significantly decreased in FZD2 siRNA-treated tumors (Figure 5B and 5C). Moreover, protein levels of stabilized active β-catenin (non-phospho β-catenin), MYC and cyclin D1 decreased as well following FZD2 blockade (*P* < 0.05; Figure 5B and 5C). These data suggest that blockade of FZD2 inhibits NB proliferation by suppressing β-catenin-dependent signaling *in vivo*.

### Blocking FZD2 activates β-catenin-independent signaling pathway components in NB xenografts

Consistent with our *in vitro* findings, we observed increased phosphorylation of PKC in SK-N-AS (2.9-fold; *P* = 0.043) and in SK-N-DZ (2.2-fold; *P* = 0.045) tumors of mice treated with FZD2 siRNA as compared to controls (Figure 5B and 5C). We did not detect any changes in expression level and activation status of JNK in both NB xenografts, in contrast to our *in vitro* findings. Activated AKT (*P* < 0.035 in SK-N-AS and SK-N-DZ) and ERK (*P* = 0.048 in SK-N-AS) were clearly increased in FZD2 siRNA-treated tumors. No evident differences in total AKT and ERK levels were observed (Figure 5B and 5C). Notably, β-catenin, MYC and PKC levels were higher in SK-N-AS tumors than in SK-N-DZ tumors (Figure 5C).

Thus, *MYCN*-unamplified SK-N-AS and *MYCN*-amplified SK-N-DZ NB xenografts respond to FZD2 blockade by downregulation of β-catenin-dependent signaling and concurrent upregulation of β-catenin-independent signaling activities.

## DISCUSSION

**Figure 6:**
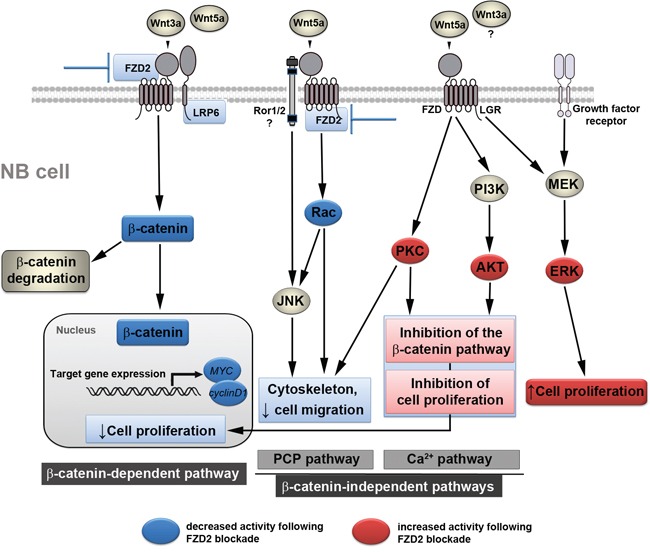
Proposed mechanism by which FZD2 blockade affects NB cell behavior Simplified schematic view of β-catenin-dependent and -independent signaling pathways and the crosstalk between each other. Blue boxes indicate inhibited signaling factors and actions, red boxes depict activated signaling factors and actions following FZD2 blockade, which were revealed in this study. Wnt3a activates the β-catenin pathway by interacting with FZD2 and LRP6. When Wnt5a acts on FZD2 it may compete with Wnt3a, thereby inhibiting the β-catenin pathway. However, Wnt5a activates the Rac/JNK pathways. Downregulation of FZD2 inhibits the β-catenin pathway and the Rac/JNK pathways in NB cells. Blocking the action of Wnt5a on these pathways by blocking FZD2 could promote the activation of other β-catenin-independent Wnt pathways. Consequently, increased availability of Wnt5a protein may interact with receptor ROR1/2 or FZD to activate the PI3K/AKT and PKC pathway, leading to the activation of target genes, which normally suppress proliferation and migration. These actions, perhaps along with modulation of the activity of other non-canonical pathways (e.g. Rac/JNK), mediate the anti-proliferative and anti-migratory effects of Wnts following FZD2 blockade. In addition, Wnts acting via FZD and growth factor receptors also activate ERK. It is unclear, whether Wnt3a may also activate a β-catenin-independent pathway and/or contributes to ERK-activation. Phosphorylation of ERK alone or alteration of the p-ERK/p-AKT ratio in turn may regulate proliferation depending on the signaling strength thus counteracting or supporting the anti-proliferative effects of FZD2 blockade.

In this study, we demonstrate that FZD2-mediates cell proliferation in two human NB cell lines with and without *MYCN* amplification and suggest a considerable degree of crosstalk between canonical and non-canonical Wnt signaling pathways in NB cells. According to our present observations, we propose a working model, in which Wnt3a and Wnt5a can form a complex with FZD2 regulating both canonical and non-canonical pathways involved in promoting cell proliferation and migration. Consequently, FZD2 blockade downregulates β-catenin-dependent signaling associated with increased activity of non-canonical pathways causing phosphorylation of PKC, AKT and ERK. The transduced signals interfere, at least in part, with the canonical Wnt/β-catenin signaling pathway leading to reduced cell proliferation (Figure 6).

The most extensively defined factor contributing to NB pathogenesis is amplification of *MYCN* oncogene. *MYCN* amplification accounts for the aggressive phenotype in a subset of children that define their tumors as high-risk and is observed in about 25% of NB cases [29]. Conversely, the majority of cases has no consistently identified molecular aberration but frequently express MYC at high levels [15]. MYC expression is dependent upon Wnt/β-catenin signaling [30]. Previous studies have implicated a potential relationship between increased Wnt/β-catenin signaling and neuroblastoma [15, 31, 32]. Likewise, we provide evidence for Wnt/FZD2 activity in high-risk NB cell lines by showing increased phospho-LRP6 and active β-catenin levels in NB cells treated with recombinant Wnt3a, consistent with canonical β-catenin signaling. Moreover, β-catenin signaling activity and expression of the β-catenin target genes MYC and cyclin D1 were coordinately reduced by FZD2 blockade associated with concomitant lower proliferation rate. These findings support basal FZD2/β-catenin pathway activity in the investigated high-risk NB cell lines. Intriguingly, our study revealed higher expression levels of FZD2, LRP6 and cyclin D1 in *MYCN*-amplified SK-N-DZ NB cells and tumors, while β-catenin (both total and active) and MYC were higher expressed in *MYCN*-unamplified SK-N-AS cells and tumors. In support of this, other studies reported upregulated β-catenin and β-catenin target genes in high-risk NB tumor tissue without *MYCN* amplification in comparison to high-risk *MYCN*-amplified tumor sections [15, 33]. Importantly, FZD2 blockade reduced β-catenin staining intensity in both NB xenografts. Together with the observed downregulation of β-catenin target genes this further strengthens our hypothesis that activation of the β-catenin pathway by FZD2 promotes growth of high-risk NBs. This is also supported by the finding that expression of β-catenin was involved in maintaining a neuroblast-like phenotype of NB cells [32]. However, based on the complexity of the involved pathways and the expression of FZD3 on both NB cell lines, we assume that the growth promoting effect of FZD2 in high-risk NB is the consequence from complex interactions between different pathways (Figure 6).

Wnt3a and Wnt5a have been shown to serve as a ligand for the FZD2 receptor. These secreted regulatory proteins may lead to activation of both canonical (Wnt3a) and non-canonical (Wnt5a) Wnt signaling pathways in cancer [23, 24, 34]. Moreover, high expression of both Wnt3 and Wnt5a has been shown in neuroblastic tumors and cell lines [35]. Findings from the present study showed that FZD2 regulates canonical Wnt3a/Wnt5a signaling in NB cells. In addition, the investigated NB cells are a source of Wnt3a and Wnt5a indicating contribution of an autocrine Wnt-signaling loop in NB growth. Likewise, autocrine Wnt signaling has been reported in non-small cell lung cancers [36]. Wnt5a is particularly interesting as some Wnt5a actions, such as blocking canonical Wnt signaling, might be expected to foster decreased tumor aggressiveness, whereas others might be expected to foster increased aggressiveness [17, 37]. Furthermore, findings by Sato et al showed that Wnt5a competes for and inhibits binding of Wnt3a to FZD2 in HeLaS3 cells, thereby suppressing the β-catenin-dependent pathway [28]. In any case, we conclude that the integration of the existing Wnt signals at the level of FZD2 promotes tumor growth in NBs. In this context, it should be noted that besides FZD, Wnt5a can also bind to the membrane-bound receptor tyrosine kinase (RTK) Ror1/2, and mediate non-canonical signal transduction [38]. Our study provides evidence that FZD2 is also involved in the control of β-catenin-independent pathways in NB. This includes the PCP pathway, in which FZD receptors activate a cascade that involves the small GTPase Rac1 and JNK, but also the Wnt/Ca^2+^ pathway, in which Wnts trigger FZD-mediated Ca^2+^ release and activate PKC [16]. Of interest, phosphorylation of JNK or PKC was not induced by stimulation with Wnt3 or Wnt5a in some NB cell lines [35]. In our cell lines, Wnts did not increase PKC phosphorylation *in vitro*, while our p-JNK *in vitro* and *in vivo* studies yielded conflicting results. However, another report showed the involvement of Wnt5a through the Wnt/Ca^2+^ signaling in the pathogenesis of NB [39]. Importantly, in *MYCN*-amplified IGR-N91 NB cells Wnt5a and PKC gene expression increased concomitantly in response to retinoic acid-induced differentiation [40]. Notably, here we show that FZD2 blockade leads to increased PKC-phosphorylation associated with downregulation of the β-catenin-dependent pathway, indicating a move from Wnt/β-catenin to Wnt/Ca^2+^ signaling in NB. Furthermore, Wnt5a-dependent Rac activation was shown in HeLaS3 cells, which was suppressed by knockdown of FZD2 [28]. In partial contrast, we observed not only Wnt5a-dependent Rac1 activation in both investigated NB cells, but also Wnt3a-dependent Rac activation in SK-N-DZ cells, indicating cell type-specific differences in Rac activation. Importantly, FZD2 blockade suppressed Wnt-dependent Rac activation and concomitant migration in both cell lines. In summary, we hypothesize that in a move away from FZD2, Wnts are turning to alternative receptors to activate non-canonical signaling pathways.

A further layer of complexity relates to the interaction of Wnt/β-catenin with RTK and the MAPK/ERK signaling pathways [41]. Evidence for a bi-directional signaling loop between Wnt/β-catenin and RTK-driven MAPK signaling pathways that serves to drive proliferation has been provided in a normal epithelial cell population. [42]. A very recent study has shown that the G-protein coupled receptor LGR5 not only regulates Wnt signaling, but also MEK/ERK signaling in NB cells and the authors speculated that Wnt signaling in SK-N-AS cells may dampen MEK/ERK signaling [43]. Consistent with this idea, our data showed that perturbation of FZD2 by siRNA leads to downregulation of LRP6 phosphorylation with concomitant upregulation of ERK phosphorylation in NB indicating a FZD2-dependent crosstalk between Wnt and ERK signaling. Alternatively, the inverse relationship of LRP6 and ERK1/2 phosphorylation may indicate a compensatory signaling response. In this model, suppression of FZD2/LRP6 pathway causes overactivation of RTK pathways, including AKT and ERK pathways that potentially oppose the antiproliferative effects of FZD2 inhibitors. Further investigation using FZD inhibitors is needed to confirm our FZD2 siRNA data and to fully understand how NB cells adapt their signaling circuitry, taking advantage of routes of feedback and crosstalk to maintain their function.

To that end, different RTKs elicit different cellular responses, yet all appear to activate RAS/ERK, SRC-family kinases and PI3K/AKT pathways. Differential responses may be obtained by modulating the relative strength of downstream pathway signaling, as has been shown for the ratio of activation of AKT and ERK pathways that distinguishes the proliferation and differentiation response in PC12 cells [44]. In this study, cyclin D knockdown cells required higher p-AKT and lower p-ERK signals to increase cyclin D protein stability and thereby make up for the reduced cyclin D translation to restore proliferation [44]. Notably, high expression of cyclin D1 occurs in approximately two thirds of NB cell lines and tumors [45]. Thus, it is tempting to speculate that the observed shift of the p-AKT/p-ERK ratio to high p-AKT/high p-ERK in FZD2 siRNA treated NBs, which was associated with reduced cyclin D1 expression levels, translates the p-ERK/p-AKT decision towards decreased proliferation of NB cells. Consequently, different routes, including different RTKs [46] and Wnt pathways, might be critical for NB proliferation.

The tumor microenvironment also substantially contributes to NB progression [47]. Highly vascular in nature, NB displays increased tumor angiogenesis, which is also related to poor patient outcomes [48]. As in most cancers, angiogenesis in NB occurs through the production of several angiogenic factors by tumor cells, which can correlate with a high-risk phenotype [49–52]. Reports from other types of cancer have also shown a direct interaction of Wnt signaling with angiogenesis [53, 54]. Likewise, the effect of FZD2 blockade on angiogenesis was obvious at animal autopsy as the control but not FZD2 siRNA-treated tumors were quite hypervascular as seen in H&E-stained tumor tissue sections. The overall anti-angiogenic effect of FZD2 blockade was further confirmed by significantly reduced levels of the endothelial cell marker CD31.

In summary, this study determines FZD2 as a likely control point for cell growth in high-risk NB tumors by regulating β-catenin-dependent and β-catenin-independent signaling pathways. FZD2 blockade by siRNA inhibited NB cell proliferation and xenograft growth, reduced cell motility, and induced a less vascularized phenotype. Thus, FZD2 represents a potential therapeutic target in FZD2-driven high-risk NB tumors.

## MATERIALS AND METHODS

### Cell lines

Human NB cell lines, SK-N-AS (MYCN single copy, chromosomal status of 1p36: loss of heterozygosity (LOH), derived from a 6 year old girl with a poorly differentiated NB) [55] and SK-N-DZ (MYCN-amplified, 1p status: no LOH, derived from a 2 year old girl with poorly differentiated NB) [55] were obtained from American Type Culture Collection (ATCC, Manassas, VA). Cells were maintained in Dulbecco's Modified Eagle's medium (DMEM, Life Technologies, Carlsbad, CA) supplemented with 0.1 mM Non-Essential Amino Acids (NEAA, Life Technologies) and fetal calf serum to a final concentration of 10% fetal bovine serum (FBS, Life Technologies) at 37°C under 5% CO_2_. Cell lines were tested for authenticity by using STR-PCR (PowerPlex 16 HS System, Promega, Madison, WI).

### Quantitative real-time RT-PCR (qRT-PCR)

NB cells and tissues were processed for PCR as described [56]. The primer sequences for human factors are as follows (sense/antisense): FZD2: 5′-TTCCACCTTCTTCACTGTCAC-3′/5′-GCCCGACA GAAAAATGATAG -3′; Wnt3a: 5′-GGCTGTTGG GCCACAGTATTCC- 3′/5′-GCTGGGCATGATCTCCACGTAG-3′; Wnt5a: 5′-ATGAACCTGCACAACAACGA- 3′/5′-CTTCTCCTTCAGGGCATCAC-3′; MYC: 5′-GGA GGAACAAGAAGATGAGG-3′/5′- TGTGCTGA TGTGGAGAC-3′; cyclin D1: 5′-AAGCTGTGCA TCTACACC-3′/5′-GATCTGTTTGTTCTCCTCC -3′. The primer sequences for mouse CD31 are as follows (sense/antisense): 5′-CAAAGAAAAGGAGGACAG-3′/5′- GATGACCACTCCAATGAC -3′.). Primer sequences for human FZD 1-10 are listed in Supplementary Table S1. LCDA Version 3.5.3 (Roche, Mannheim, Germany) was used for PCR data analysis. Relative quantification of the signals was performed by normalizing the signals of the different genes to β2-microglobulin as described [56, 57].

### Protein isolation and western blotting

NB cells were seeded in 100 mm plates, one portion of cells were left untreated and one portion of cells were starved for 24 h before incubation with 100 ng/ml recombinant Wnt3a and Wnt5a protein for 15 min. Cell lysates were prepared [58] and 25 μg/lane were separated by 8/12% SDS-PAGE prior to electrophoretic transfer onto Amersham Protan Supported 0.2 μm Nitrocellulose membrane (GE Healthcare, Buckinghamshire, UK). The blots were probed with antibodies against FZD2 (Thermo Fisher Scientific, Waltham, MA), phospho-LRP6 (Ser1490) (Cell Signaling Technology, Danvers, MA), β-catenin (Abcam, Cambridge, UK), non-phospho β-catenin (Ser33/37/Thr41), MYC, cyclin D1, phospho-PKC (pan) (βII Ser660), JNK, phospho-JNK (Thr183/Tyr185), AKT, phospho-AKT (Ser473), ERK1/2 and phospho-44/42 ERK1/2(Thr202/Tyr204) (Cell Signaling Technology) before incubation with horseradish peroxidase–conjugated secondary antibodies (GE Healthcare). GAPDH-HRP staining (Sigma-Aldrich, St. Louis, MO) was used as loading control. Proteins were immunodetected by chemiluminescence (Ace Glow, Peqlab, Erlangen, Germany), scanned using FUSION-FX7 (Vilber Lourmat, Marne-la-Vallée, France) and quantified by Fusion-CAPT-Software 16.07 (Vilber Lourmat).

### Small interfering RNA knockdown in NB cell lines *in vitro*

Three siRNAs for each target gene and two scrambled sequences were designed (synthesized from Eurofins Genomics AT, Vienna, Austria) and examined *in vitro*. The sequence of the siRNA targeting FZD2 was: 5′- CCACGTACTTGGTAGACAT -3′; the sequence of the scrambled siRNA was: 5′-GAAGCAGCACGACTTCTTCTT-3′. Screening experiments were performed with *in vitro* transcribed siRNAs [59] and knockdown efficiency was assessed by qRT-PCR and Western blotting. For knockdown experiments *in vitro*, SK-N-AS and SK-N-DZ NB cells that express FZD2 were cultured in DMEM containing 10% FBS to 60% confluency. Cells were rinsed with PBS, refed with serum-free medium and then transfected with 100 nM siRNA targeting FZD2 or scrambled siRNA using Lipofectamine and Plus reagent (Life Technologies) according to the manufacturer's protocol. At 24 h after transfection, NB cells were starved for further 24 h before incubation with 100 ng/ml recombinant Wnt3a and Wnt5a protein for 15 min. Untreated cells served as additional controls. Experiments were performed in triplicates. Next protein was isolated and analyzed by Western blotting as described [60].

### Cell proliferation assay

Human SK-N-AS and SK-N-DZ NB cells were seeded in 96-well plates at a density of 1×10^4^ cells/well in DMEM supplemented with 10% FBS. After 24 h cells were transfected with 100 nM FZD2 siRNA or scrambled siRNA using Lipofectamine and Plus reagent (Thermo Fisher Scientific). After another 24 h culture medium was replaced with DMEM with 1% FBS with or without 100 ng/ml recombinant Wnt3a or Wnt5a protein (R&D Systems, McKinley Place, MN). After 24, 48 and 72 h cell proliferation was determined using the colorimetric-based WST-1 reagent (Roche Diagnostics, Indianapolis, IN) according to the manufacturer's protocol.

### Migration assay

NB cells (1×10^5^ in 1 ml DMEM with 10% FBS) were added to the top of each Boyden migration chamber (8 μm, 12-well plate format; BD Biosciences, Palo Alto, CA). After 24 h cells were transfected with 100 nM FZD2 siRNA or scrambled siRNA using Lipofectamine and Plus reagent. After 24 h cells were incubated with 100 ng/ml recombinant Wnt3a or Wnt5a protein in DMEM with 1% FBS. After 48 h, medium was removed and membranes were washed twice with phosphate buffered saline (PBS). Cells from the upper side of the membrane were removed with cotton swabs. The membranes were excised using a scalpel, inverted and transferred to a PBS filled tissue culture well. Membranes were then fixed in methanol for 10 min at −20°C. After washing in PBS, membranes were stained with 1 μg/ml 4′-6-Diamidino-2-phenylindole (DAPI) in PBS for 10 min at room temperature and washed again in PBS. Membranes were then embedded in Cityfluor (Cityfluor, Leicester, UK) on glass slides. Representative sectors of migrated cancer cells were counted under a fluorescence microscope. Each experiment was performed in triplicate.

### Rac1 activation

NB cells were seeded in 6-well plates. After 24 h cells were transfected with FZD2 and scrambled siRNA. 24 hours after transfection cells were starved for 24 h. Cells were stimulated with 100 ng/ml recombinant Wnt3a and Wnt5a protein for 30 min and Rac1 activity was then measured with G-LISA (colorimetric format, Cytoskeleton, Denver, CO) according to the manufacturer's protocol.

### Analysis of FZD2 siRNA treatment *in vivo*

The experiments performed in this study were approved by the Institutional Animal Care and Use Committee at the Medical University of Vienna. Pathogen-free immune-deficient male athymic *nu/nu* (nude) mice (Charles River, Sulzfeld, Germany), 5 weeks of age, were weighed and coded and randomly assigned to experimental groups of *n* = 8. Mice were anesthetized (ketamine hydrochloride/xylazine at 55/7.5 mg/kg, i.p.), and 4×10^6^ SK-N-AS or 8×10^6^ SK-N-DZ cells/150 μl PBS were injected s.c. into their left flank [61]. In the present study, mice developed human NBs of similar weight at 10 days. FZD2 siRNA and scrambled siRNA (control) treatment was started on day 10 at a dose of 10 μg/injection intratumorally, and cycled every three days. All animals were sacrificed on day 24, and tumors were isolated and weighed. One portion of the tissue was processed for paraffin embedding, and the remainder was processed for real-time qRT-PCR and Western blotting.

### Histology and immunohistochemistry

Paraffin-embedded serial sections were rehydrated, incubated in 5% H_2_O_2_ to block endogenous peroxidase activity and antigens were stained with hematoxylin and eosin (H&E), or subjected to immunohistochemical analysis for Ki67 antibody (tumor proliferation assay; Dako, Glostrup, Denmark) to evaluate the density of proliferating cells [58] and total β-catenin (Abcam). Ki67 and β-catenin primary antibodies were detected with biotinylated secondary antibody (Vector Laboratories, Burlingame, CA) and peroxidase conjugated streptavidin (Dako), developed with 3,3′-diaminobenzidine (Vector Laboratories), counterstained with haemalaun, dehydrated and mounted in DPX (Merck, Darmstadt, Germany) and digitalized images were generated with a Nikon Eclipse 80i (Tokyo, Japan) microscope and analyzed using NIS Elements imaging software (Nikon). Results are expressed as relative percentage of Ki67–positive cells per field.

### Statistical analysis

Differences between two groups were studied using the two-sided Student's *t*-test. When more than two groups were compared, we performed analysis of variance (ANOVA) followed by post hoc tests (Dunnet, Bonferroni). All statistical tests were two-sided. Data are expressed as means ± the standard deviation (SD). *P* values of < 0.05 were considered to indicate statistical significance. Statistical tests were performed with the use of Statistical Package for the Social Sciences (SPSS) software (version 22.0, SPSS Inc., Chicago, IL).

## SUPPLEMENTARY DATA



## References

[1] Van Noesel MM, Versteeg R (2004). Pediatric neuroblastomas: genetic and epigenetic ‘Danse Macabre’. Gene.

[2] Matthay KK, Villablanca JG, Seeger RC, Stram DO, Harris RE, Ramsay NK, Swift P, Shimada H, Black CT, Brodeur GM, Gerbing RB, Reynolds CP, Grp CC (1999). Treatment of high-risk neuroblastoma with intensive chemotherapy, radiotherapy, autologous bone marrow transplantation, and 13-cis-retinoic acid. New England Journal of Medicine.

[3] Brodeur GM (2003). Neuroblastoma: biological insights into a clinical enigma. Nature reviews Cancer.

[4] Huang M, Weiss WA (2013). Neuroblastoma and MYCN. Cold Spring Harbor perspectives in medicine.

[5] Cheung NK, Dyer MA (2013). Neuroblastoma: developmental biology, cancer genomics and immunotherapy. Nature reviews Cancer.

[6] Polakis P (2012). Wnt signaling in cancer. Cold Spring Harbor perspectives in biology.

[7] Chenn A, Walsh CA (2002). Regulation of cerebral cortical size by control of cell cycle exit in neural precursors. Science.

[8] Zechner D, Fujita Y, Hulsken J, Muller T, Walther I, Taketo MM, Crenshaw EB, Birchmeier W, Birchmeier C (2003). beta-catenin signals regulate cell growth and the balance between progenitor cell expansion and differentiation in the nervous system. Developmental Biology.

[9] Lee HY, Kleber M, Hari L, Brault V, Suter U, Taketo MM, Kemler R, Sommer L (2004). Instructive role of Wnt/beta-catenin in sensory fate specification in neural crest stem cells. Science.

[10] Hecht A, Kemler R (2000). Curbing the nuclear activities of beta-catenin - Control over Wnt target gene expression. Embo Reports.

[11] Ilyas M (2005). Wnt signalling and the mechanistic basis of tumour development. Journal of Pathology.

[12] Milovanovic T, Planutis K, Nguyen A, Marsh JL, Lin F, Hope C, Holcombe RF (2004). Expression of Wnt genes and frizzled 1 and 2 receptors in normal breast epithelium and infiltrating breast carcinoma. International journal of oncology.

[13] Merle P, Kim M, Herrmann M, Gupte A, Lefrancois L, Califano S, Trepo C, Tanaka S, Vitvitski L, de la Monte S, Wands JR (2005). Oncogenic role of the frizzled-7/beta-catenin pathway in hepatocellular carcinoma. Journal of Hepatology.

[14] Ueno K, Hiura M, Suehiro Y, Hazama S, Hirata H, Oka M, Imai K, Dahiya R, Hinoda Y (2008). Frizzled-7 as a potential therapeutic target in colorectal cancer. Neoplasia.

[15] Liu X, Mazanek P, Dam V, Wang Q, Zhao H, Guo R, Jagannathan J, Cnaan A, Maris JM, Hogarty MD (2008). Deregulated Wnt/beta-catenin program in high-risk neuroblastomas without MYCN amplification. Oncogene.

[16] Niehrs C (2012). The complex world of WNT receptor signalling. Nature reviews Molecular cell biology.

[17] Kikuchi A, Yamamoto H, Sato A, Matsumoto S (2011). New Insights into the Mechanism of Wnt Signaling Pathway Activation. International Review of Cell and Molecular Biology.

[18] De A (2011). Wnt/Ca2+ signaling pathway: a brief overview. Acta biochimica et biophysica Sinica.

[19] Dissanayake SK, Wade M, Johnson CE, O′Connell MP, Leotlela PD, French AD, Shah KV, Hewitt KJ, Rosenthal DT, Indig FE, Jiang Y, Nickoloff BJ, Taub DD, Trent JM, Moon RT, Bittner M (2007). The Wnt5A/protein kinase C pathway mediates motility in melanoma cells via the inhibition of metastasis suppressors and initiation of an epithelial to mesenchymal transition. The Journal of biological chemistry.

[20] Anastas JN, Kulikauskas RM, Tamir T, Rizos H, Long GV, von Euw EM, Yang PT, Chen HW, Haydu L, Toroni RA, Lucero OM, Chien AJ, Moon RT (2014). WNT5A enhances resistance of melanoma cells to targeted BRAF inhibitors. The Journal of clinical investigation.

[21] Holcombe RF, Marsh JL, Waterman ML, Lin F, Milovanovic T, Truong T (2002). Expression of Wnt ligands and Frizzled receptors in colonic mucosa and in colon carcinoma. Journal of Clinical Pathology-Molecular Pathology.

[22] Rhee CS, Sen M, Lu DS, Wu C, Leoni L, Rubin J, Corr M, Carson DA (2002). Wnt and frizzled receptors as potential targets for immunotherapy in head and neck squamous cell carcinomas. Oncogene.

[23] Bazhin AV, Tambor V, Dikov B, Philippov PP, Schadendorf D, Eichmuller SB (2010). cGMP-phosphodiesterase 6, transducin and Wnt5a/Frizzled-2-signaling control cGMP and Ca(2+) homeostasis in melanoma cells. Cellular and molecular life sciences.

[24] Li C, Chen H, Hu L, Xing Y, Sasaki T, Villosis MF, Li J, Nishita M, Minami Y, Minoo P (2008). Ror2 modulates the canonical Wnt signaling in lung epithelial cells through cooperation with Fzd2. BMC molecular biology.

[25] Prasad CP, Mohapatra P, Andersson T (2015). Therapy for BRAFi-Resistant Melanomas: Is WNT5A the Answer?. Cancers.

[26] Yun MS, Kim SE, Jeon SH, Lee JS, Choi KY (2005). Both ERK and Wnt/beta-catenin pathways are involved in Wnt3a-induced proliferation. Journal of Cell Science.

[27] Shojima K, Sato A, Hanaki H, Tsujimoto I, Nakamura M, Hattori K, Sato Y, Dohi K, Hirata M, Yamamoto H, Kikuchi A (2015). Wnt5a promotes cancer cell invasion and proliferation by receptor-mediated endocytosis-dependent and -independent mechanisms, respectively. Scientific reports.

[28] Sato A, Yamamoto H, Sakane H, Koyama H, Kikuchi A (2010). Wnt5a regulates distinct signalling pathways by binding to Frizzled2. The EMBO journal.

[29] Maris JM (2010). Medical Progress: Recent Advances in Neuroblastoma. New England Journal of Medicine.

[30] Bellmeyer A, Krase J, Lindgren J, LaBonne C (2003). The protooncogene c-Myc is an essential regulator of neural crest formation in Xenopus. Developmental Cell.

[31] Coulon A, Flahaut M, Muhlethaler-Mottet A, Meier R, Liberman J, Balmas-Bourloud K, Nardou K, Yan P, Tercier S, Joseph JM, Sommer L, Gross N (2011). Functional Sphere Profiling Reveals the Complexity of Neuroblastoma Tumor-Initiating Cell Model. Neoplasia.

[32] Zhi F, Gong GM, Xu Y, Zhu Y, Hu D, Yang YL, Hu YQ (2012). Activated beta-catenin Forces N2A Cell-derived Neurons Back to Tumor-like Neuroblasts and Positively Correlates with a Risk for Human Neuroblastoma. International journal of biological sciences.

[33] Jansen SR, Holman R, Hedemann I, Frankes E, Elzinga CRS, Timens W, Gosens R, de Bont ES, Schmidt M (2015). Prostaglandin E-2 promotes MYCN non-amplified neuroblastoma cell survival via beta-catenin stabilization. Journal of Cellular and Molecular Medicine.

[34] Bhanot P, Brink M, Samos CH, Hsieh JC, Wang YS, Macke JP, Andrew D, Nathans J, Nusse R (1996). A new member of the frizzled family from Drosophila functions as a Wingless receptor. Nature.

[35] Revet I, Huizenga G, Koster J, Volckmann R, van Sluis P, Versteeg R, Geerts D (2010). MSX1 induces the Wnt pathway antagonist genes DKK1, DKK2, DKK3, and SFRP1 in neuroblastoma cells, but does not block Wnt3 and Wnt5A signalling to DVL3. Cancer letters.

[36] Akiri G, Cherian MM, Vijayakumar S, Liu G, Bafico A, Aaronson SA (2009). Wnt pathway aberrations including autocrine Wnt activation occur at high frequency in human non-small-cell lung carcinoma. Oncogene.

[37] Stewart DJ (2014). Wnt Signaling Pathway in Non-Small Cell Lung Cancer. Jnci-Journal of the National Cancer Institute.

[38] Nishita M, Enomoto M, Yamagata K, Minami Y (2010). Cell/tissue-tropic functions of Wnt5a signaling in normal and cancer cells. Trends in Cell Biology.

[39] Blanc E, Goldschneider D, Douc-Rasy S, Benard J, Raguenez G (2005). Wnt-5a gene expression in malignant human neuroblasts. Cancer letters.

[40] Blanc E, Le Roux G, Benard J, Raguenez G (2005). Low expression of Wnt-5a gene is associated with high-risk neuroblastoma. Oncogene.

[41] Bikkavilli RK, Malbon CC (2009). Mitogen-activated protein kinases and Wnt/beta-catenin signaling: Molecular conversations among signaling pathways. Communicative & integrative biology.

[42] Georgopoulos NT, Kirkwood LA, Southgate J (2014). A novel bidirectional positive-feedback loop between Wnt-beta-catenin and EGFR-ERK plays a role in context-specific modulation of epithelial tissue regeneration. Journal of Cell Science.

[43] Vieira GC, Chockalingam S, Melegh Z, Greenhough A, Malik S, Szemes M, Park JH, Kaidi A, Zhou L, Catchpoole D, Morgan R, Bates DO, Gabb PD, Malik K (2015). LGR5 regulates pro-survival MEK/ERK and proliferative Wnt/beta-catenin signalling in neuroblastoma. Oncotarget.

[44] Chen JY, Lin JR, Cimprich KA, Meyer T (2012). A Two-Dimensional ERK-AKT Signaling Code for an NGF-Triggered Cell-Fate Decision. Molecular Cell.

[45] Molenaar JJ, van Sluis P, Boon K, Versteeg R, Caron HN (2003). Rearrangements and increased expression of cyclin D1 (CCND1) in neuroblastoma. Genes, chromosomes & cancer.

[46] Palacios-Moreno J, Foltz L, Guo AL, Stokes MP, Kuehn ED, George L, Comb M, Grimes ML (2015). Neuroblastoma Tyrosine Kinase Signaling Networks Involve FYN and LYN in Endosomes and Lipid Rafts. Plos Computational Biology.

[47] Borriello L, Seeger RC, Asgharzadeh S, DeClerck YA (2015). More than the genes, the tumor microenvironment in neuroblastoma. Cancer letters.

[48] Meitar D, Crawford SE, Rademaker AW, Cohn SL (1996). Tumor angiogenesis correlates with metastatic disease, N-myc amplification, and poor outcome in human neuroblastoma. Journal of Clinical Oncology.

[49] Backman U, Svensson A, Christofferson R (2002). Importance of vascular endothelial growth factor A in the progression of experimental neuroblastoma. Angiogenesis.

[50] Fakhari M, Pullirsch D, Paya K, Abraham D, Hofbauer R, Aharinejad S (2002). Upregulation of vascular endothelial growth factor receptors is associated with advanced neuroblastoma. Journal of Pediatric Surgery.

[51] Fakhari M, Pullirsch D, Abraham D, Paya K, Hofbauer R, Holzfeind P, Hofmann M, Aharinejad S (2002). Selective upregulation of vascular endothelial growth factor receptors neuropilin-1 and -2 in human neuroblastoma. Cancer.

[52] Meister B, Grunebach F, Bautz F, Brugger W, Fink FM, Kanz L, Mohle R (1999). Expression of vascular endothelial growth factor (VEGF) and its receptors in human neuroblastoma. European Journal of Cancer.

[53] Zhang X, Gaspard JP, Chung DC (2001). Regulation of vascular endothelial growth factor by the Wnt and K-ras pathways in colonic neoplasia. Cancer Research.

[54] Hu J, Dong AW, Fernandez-Ruiz V, Shan JJ, Kawa M, Martinez-Anso E, Prieto J, Qian C (2009). Blockade of Wnt Signaling Inhibits Angiogenesis and Tumor Growth in Hepatocellular Carcinoma. Cancer Research.

[55] Thompson PM, Maris JM, Hogarty MD, Seeger RC, Reynolds CP, Brodeur GM, White PS (2001). Homozygous deletion of CDKN2A (p16INK4a/p14ARF) but not within 1p36 or at other tumor suppressor loci in neuroblastoma. Cancer Research.

[56] Zins K, Thomas A, Lucas T, Sioud M, Aharinejad S, Abraham D (2013). Inhibition of Stromal PlGF Suppresses the Growth of Prostate Cancer Xenografts. International Journal of Molecular Sciences.

[57] Paulus P, Stanley ER, Schafer R, Abraham D, Aharinejad S (2006). Colony-stimulating factor-1 antibody reverses chemoresistance in human MCF-7 breast cancer xenografts. Cancer Research.

[58] Zins K, Gunawardhana S, Lucas T, Abraham D, Aharinejad S (2013). Targeting Cdc42 with the small molecule drug AZA197 suppresses primary colon cancer growth and prolongs survival in a preclinical mouse xenograft model by downregulation of PAK1 activity. Journal of Translational Medicine.

[59] Sorensen DR, Leirdal M, Sioud M (2003). Gene silencing by systemic delivery of synthetic siRNAs in adult mice. Journal of Molecular Biology.

[60] Zins K, Abraham D, Sioud M, Aharinejad S (2007). Colon cancer cell-derived tumor necrosis factor-alpha mediates the tumor growth-promoting response in macrophages by up-regulating the colony-stimulating factor-1 pathway. Cancer Research.

[61] Abraham D, Zins K, Sioud M, Lucas T, Schafer R, Stanley ER, Aharinejad S (2010). Stromal cell-derived CSF-1 blockade prolongs xenograft survival of CSF-1-negative neuroblastoma. International Journal of Cancer.

