# Supplementary material for: Frizzled2 signaling regulates growth of high-risk neuroblastomas by interfering with β-catenin-dependent and β-catenin-independent signaling pathways
Source: Oncotarget. 2016 Jun 15;7(29):46187–202. doi: 10.18632/oncotarget.10070 (PMC5216790; doi:10.18632/oncotarget.10070)
Supplement: Supplementary file 1 [file oncotarget-07-46187-s001.pdf]

Frizzled2 signaling regulates growth of high-risk neuroblastomas by interfering with β-catenin-dependent and β-catenin-independent signaling pathways

SUPPLEMENTARY DATA

EXPRESSION OF FZD1-10 MRNAS IN HUMAN NB CELL LINES

Supplementary material

NB cells and tissues were processed for qRT-PCR as described in the manuscript. The primer sequences for human factors are as follows (sense/antisense):

Results

We investigated the mRNA expression of FZD1-10 in SK-N-AS and SK-N-DZ NB cell lines by quantitative RT-PCR. FZD2 mRNA in both cell lines was the highest, followed by FZD3, while expression of other FZDs was low. SK-N-DZ cells expressed significantly higher FZD2 and FZD3 levels than SK-N-AS cells. (Supplemental Figure 1).

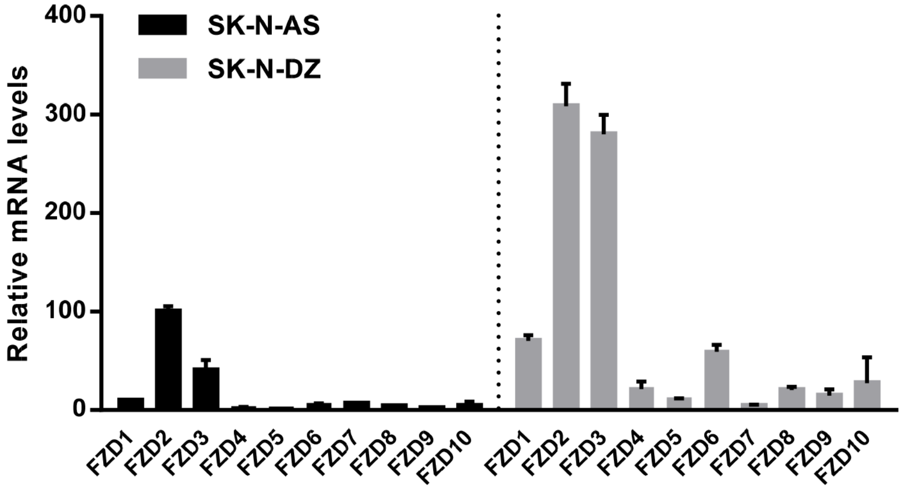

Supplementary Figure S1: Characterization of gene expression in *MYCN*-unamplified SK-N-AS and *MYCN*-amplified SK-N-DZ NB cells. Graph shows the results of qRT-PCR for FZD1-10 performed on mRNA from human SK-N-AS and SK-N-DZ NB cells. FZD1-10 mRNA expression is different between SK-N-AS and SK-N-DZ cells. Graphs represent the mean of 3 independent replicates ± the standard deviation.

Supplementary Table S1: Primer sequences for human FZD 1-10 (sense/antisense)

Gene	Primer sense 5′-3′	Primer antisense 5′-3′
FZD1	GTGAGCCGACCAAGGTGTAT	CAGCCGGACAAGAAGATGAT
FZD2	TTCCACCTTCTTCACTGTCAC	GCCCGACAGAAAAATGATAG
FZD3	TGAGTGTTCGAAGCTCATGG	ATCACGCACATGCAGAAAAG
FZD4	AACCTCGGCTACAACGTGAC	GTTGTGGTCGTTCTGTGGTG
FZD5	TGCTACCAGCCGTCCTTCAGT	CCATGCCGAAGAAGTAGACCAG
FZD6	ATTTTGGTGTCCAAGGCATC	TATTGCAGGCTGTGCTATCG
FZD7	GTGCAGTGTTCTCCCGAACT	GAACGGTAAAGAGCGTCGAG
FZD8	TCTTGTCGCTCACATGGTTC	TGTAGAGCACGGTGAACAGG
FZD9	CGCTGGTCTTCCTACTGCTC	AGAAGACCCCGATCTTGACC
FZD10	GCGGTGAAGACCATCCTG	GCACGGTGTACAGCACAGAG
